# The epidemiological situation of tuberculosis in Spain according to surveillance and hospitalization data, 2012–2020

**DOI:** 10.1371/journal.pone.0295918

**Published:** 2024-01-02

**Authors:** Teresa Pedraz, Laura Herrera, Maria C. Vazquez, Oriana Ramírez-Rubio, Rosa Cano, Zaida Herrador, on behalf of the Spanish Working Group on Tuberculosis

**Affiliations:** 1 Department of Preventive Medicine, University Hospital La Paz, Madrid, Spain; 2 Department of Bacteriology, National Centre of Microbiology, Instituto de Salud Carlos III, Majadahonda, Spain; 3 CIBER in Epidemiology and Public Health (CIBERESP), Madrid, Spain; 4 SG Prevención, Promoción y Educación para la Salud, Direccion General de Salud Pública, Consejeria de Sanidad, Madrid, Spain; 5 Division for HIV, STI, Viral Hepatitis and TB Control, Ministry of Health, Madrid, Spain; 6 National Center of Epidemiology, Instituto de Salud Carlos III, Madrid, Spain; Instituto Politecnico Nacional, MEXICO

## Abstract

**Background:**

Before the COVID-19 pandemic, tuberculosis (TB) was the leading cause of death from a single infectious agent. In Spain, TB notifications are registered through the National Epidemiological Surveillance Network (RENAVE). The Minimum Basic Data Set (CMBD) provides information on TB hospital discharges. This study aims to assess both registries to complete the picture of TB in order to improve national control strategies and make further progress toward its elimination.

**Methods:**

A retrospective study was performed considering CMBD´s hospital discharges with TB as first diagnosis and notifications to RENAVE between 2012 and 2020. After describing the records of both systems and their differences by using descriptive and multivariate analysis, annual incidences rates were calculated in order to evaluate temporal trends and geographical patters.

**Results:**

According to the CMBD database, there were 29,942 hospitalizations due to TB (65% pulmonary forms and 66% male) during the study period. RENAVE collected 44,520 reported cases, mostly males (62%) with pulmonary forms (72%). Young children were similar in both groups, showing the high frequency of hospitalization in this group. Almost all autonomous communities showed a downward trend, especially Asturias. Hospitalizations in 2020 were analyzed by month separately, and comparing with previous years, the impact of the COVID-19 pandemic can be seen.

**Conclusions:**

A decreasing trend on TB incidence was observed in Spain since 2012, although this trend might change after COVID-19 pandemic. The analysis of both databases, CMBD and RENAVE, has contributed to improve our knowledge of TB in Spain and will help improve the control of this disease.

## Introduction

Tuberculosis (TB) is an infectious disease caused by *Mycobacterium tuberculosis*, a highly aerobic bacillus that most commonly colonizes the lungs. Before the COVID-19 pandemic, TB was the leading cause of death from a single infectious agent. According to the World Health Organization (WHO) Global Tuberculosis Report 2022 [1], COVID-19 has reinforced the historical allies of TB: poverty, social exclusion, overcrowding, institutionalization, concomitant pathologies or barriers to access to health services [2]. Globally, an estimated 10.6 million people (95% UI: 9.9–11 million) fell ill with TB in 2021, an increase of 4.5% from 10.1 million in 2020. The number of deaths from TB have also increased between 2019 and 2021, reversing years of decline between 2005 and 2019 [1].

Ending the TB epidemic by 2030 is one of the United Nations (UN) Sustainable Development Goals (SDGs), while the WHO End TB Strategy aims to reduce deaths by 90% and TB incidence by 80% in 2030 compared to 2015 [3]. Several challenges continue to slow the progress towards these goals, i.e., the shortage of funds, the difficulties posed by latent infections, and drug-resistant TB, a problem that is expanding and has led the WHO to modify the clinical guidelines in 2022 with new regimens that include bedaquiline or delamanid [4, 5].

According to the European Centre for Disease Prevention and Control (ECDC), Spain is considered to be among the countries with a low incidence (7.8 cases/100,000 inhabitants in 2020 [2], with a decreasing trend maintained in the last decade [2, 6]. Since 1904, the reporting of pulmonary TB has been mandatory in this country [7]. However, until 1995, it was only mandatory to notify the total number of pulmonary TB cases at state level. After the creation of the National Network of Epidemiological Surveillance (RENAVE in Spanish) in 1995, the rest of clinical forms also became notifiable, and individualized notification was also established along with an epidemiological survey of the cases [8, 9].

There are other sources that provide useful information on TB epidemiology in Spain. The minimum basic data set (CMBD in Spanish) has collected data on TB hospital discharges since 1997. Up to date, several authors have explored this data source to study the epidemiology of infectious diseases in some regions or to complement information on tuberculosis cases [10–13]. This study aims at describing TB hospital discharges from 2012 to 2020 and compare them with the TB cases reported to RENAVE, seeking a more complete picture of the TB situation in Spain. If public health surveillance is "information for action," the better we know the epidemiology of TB, the better equipped we will be to improve TB control strategies and make further progress toward its elimination.

## Methods

### Study design

Retrospective descriptive study using CMBD information on TB related hospitalization, and TB cases reported to RENAVE between January 1st, 2012 and December 31st, 2020 in Spain.

### Data sources and study population

#### CMBD and RAE-CMBD

The CMBD is a set of clinical-administrative data of hospitalizations implemented since 1997 that collects sociodemographic, clinical and administrative patient data from the National Health System (NHS) discharge reports [14]. Spanish NHS provides free medical care to 99.5% of the Spanish population. Since 2005, CMBD also has a gradual coverage from private hospitals. In 2016, the CMBD was expanded and renamed RAE-CMBD as a new data model that included any contact established with the Spanish specialized health care network [15].

All CMBD’s hospital discharges with TB diagnosis in first diagnostic position between 2012 and 2020 were analyzed. These nine years include the change from CMBD to RAE-CMBD, as well as the change of coding from ICD-9 to ICD-10. Therefore, it was necessary to homogenize both datasets and unify them. All ICD-9 and ICD-10 codes used are detailed in S1 Table.

Sociodemographic and administrative data were collected from each hospitalization (sex, age, date of admission, date of discharge and autonomous community of admission, duration and costs of hospitalization, type of discharge, type of tuberculosis, HIV coinfection, and exitus). To identify those TB hospitalizations related to HIV, the following codes were searched for in any diagnostic positions: in ICD-9: V08, 042, 079.53 and in ICD-10: B20, Z21.

The classification of pulmonary/extrapulmonary tuberculosis was made following the RENAVE definition [4]. The length of hospitalization was categorized whether the hospitalization exceeded 15 days or not. Costs were calculated using diagnostic cost groups, based, in turn, on the diagnostic related groups (DRG), age, sex, and resource consumption [13]. DRG were categorized in two groups, depending on whether they were lower or higher than the mean average.

Finally, to compare both data sources it was necessary to identify those patients treated on more than one occasion, as many admissions are possible for a single patient. For this reason, we created a new variable for "successive episode" based on shared patient identifier, hospital, sex and date of birth, but different date of discharge. To estimate hospitalization rates per 100,000 inhabitants, and for comparisons with RENAVE data, the records identified as "successive" were excluded, and only the first episode of each patient was considered.

#### RENAVE

RENAVE was established in 1995 with the aim of collecting and analyzing epidemiological information that allows detecting problems that pose a public health threat. The regional surveillance systems of the autonomous communities (CCAA in Spanish) report individually data on suspected, probable and confirmed cases of tuberculosis to the National Epidemiology Center (CNE in Spanish) through RENAVE. In addition, once a year, the CCAA complete the information with the rest of the variables included in the epidemiological survey. The notification is made following guidelines and protocols agreed by all the members of RENAVE (representatives of the CCAA, Carlos III Health Institute and the Ministry of Health). Case definitions in this protocol are based on the EU case definitions, as published in the *Official Journal of the European Union* (Commission Implementing Decision (EU) 2018/945) [16]. Collected information includes CCAA and province (residence and place of infection), date of birth, sex, age, case classification, date for statistics (symptoms onset date, when available, or the closest one), type of tuberculosis (pulmonary/extrapulmonary), HIV status, hospitalization and exitus.

#### INE

Both databases are comprehensive and cover the entire national territory. Population at risk was obtained from the National Institute for Statistics (INE) [17]. All population officially residing in Spain were included in the denominator. The average number of hospitalizations per year, annual hospital admissions rate and annual incidence rates (per 100,000 per year) were calculated by CCAA, age group and sex. Information on deaths with TB as main cause recorded during the study period were also extracted from INE mortality data.

### Data analysis

Main study outcomes were the increased risk related to age group, sociodemographic and clinical characteristics associated with pulmonary forms, the average number of hospitalizations per year and CCAA and its temporal trend and moving averages.

The CMBD and RENAVE patients´ characteristics were described according to sex, age and whether it was a pulmonary form or not. We used frequencies, percentages, mean ± standard deviation (SD), medians and interquartile range (IQR) to summarize data. Age was categorized into five groups: 0–4, 5–14, 15–44, 45–64 and 65 or older, to provide a more detailed view of early childhood, children, young adults, adults and the elderly, respectively. Suspected, probable and confirmed cases from RENAVE were included in the analysis.

Differences in proportions between groups were assessed using χ2 and Student’s t tests for qualitative and quantitative variables. To estimate the increased risk related to age group by clinical forms of tuberculosis, ANOVA test was performed. We used two-sided tests and p < 0.05 was considered significant.

Patient characteristics associated with pulmonary forms within CMBD cases were explored with bivariate analysis. Those variables with p value below 0.25 were included in a logistic regression model. The crude and adjusted odds ratio and its 95% confidence interval were estimated.

To assess temporal and geographical patterns, the average number of hospitalizations per year and CCAA were computed. Temporal trends were calculated using linear and Jointpoint regression analysis (Jointpoint software version 4.9.1.0, National Cancer Institute, Bethseda, Maryland) [18]. This method identifies the year(s) when a trend change is produced by calculating the annual percentage change (APC) in rates with corresponding 95% confidence intervales between trend-change points. It also estimates the average annual percentage change (AAPC) in the whole period studied. When there are no join points (i.e., no changes in trend), APC is constant, and thus equals the AAPC. Otherwise, the whole period is segmented by the points with trend change.

Regarding analysis of spatial patterns, only data from 2012 to 2018 were analyzed, as 2018 was the last year with data consolidated by the CCAA in the RENAVE, and thus comparable with the CMBD data. Annual hospitalization and reporting rates were distributed into 6 categories based on the average percentage change or reporting rate depending on whether it was CMBD or RENAVE data, respectively. Categories were defined based on underlying joinpoint model that best fits the data.

To assess the possible impact of the COVID-19 pandemic, a time series analysis with data from 2020 was performed. Using CMBD, the centered simple moving averages (SMA) of the hospitalization rate per 100,000 inhabitants between 2012 and 2019 were calculated and compared with those of 2020. A SMA is an arithmetic moving average calculated by adding recent number of cases by month and then dividing that figure by the number of time periods in the calculation average (n = 8 in this particular case). To build the prediction limits, we used the mean squared deviation (MSD) following the formula: $SMA+\frac{}{-}1.96\;x\;\sqrt{MSD}.$

Maps were created with Mapchart.net/spain.html. This work is licensed under a Creative Commons Attribution-ShareAlike 4.0 International License (CC BY-SA 4.0). For the rest of the analysis, Stata Version 17.0 software and Microsoft Excel 2019 MAO 64-bit were used.

## Results

### Hospitalizations due to tuberculosis in Spain from 2012 to 2020

**Fig 1:**
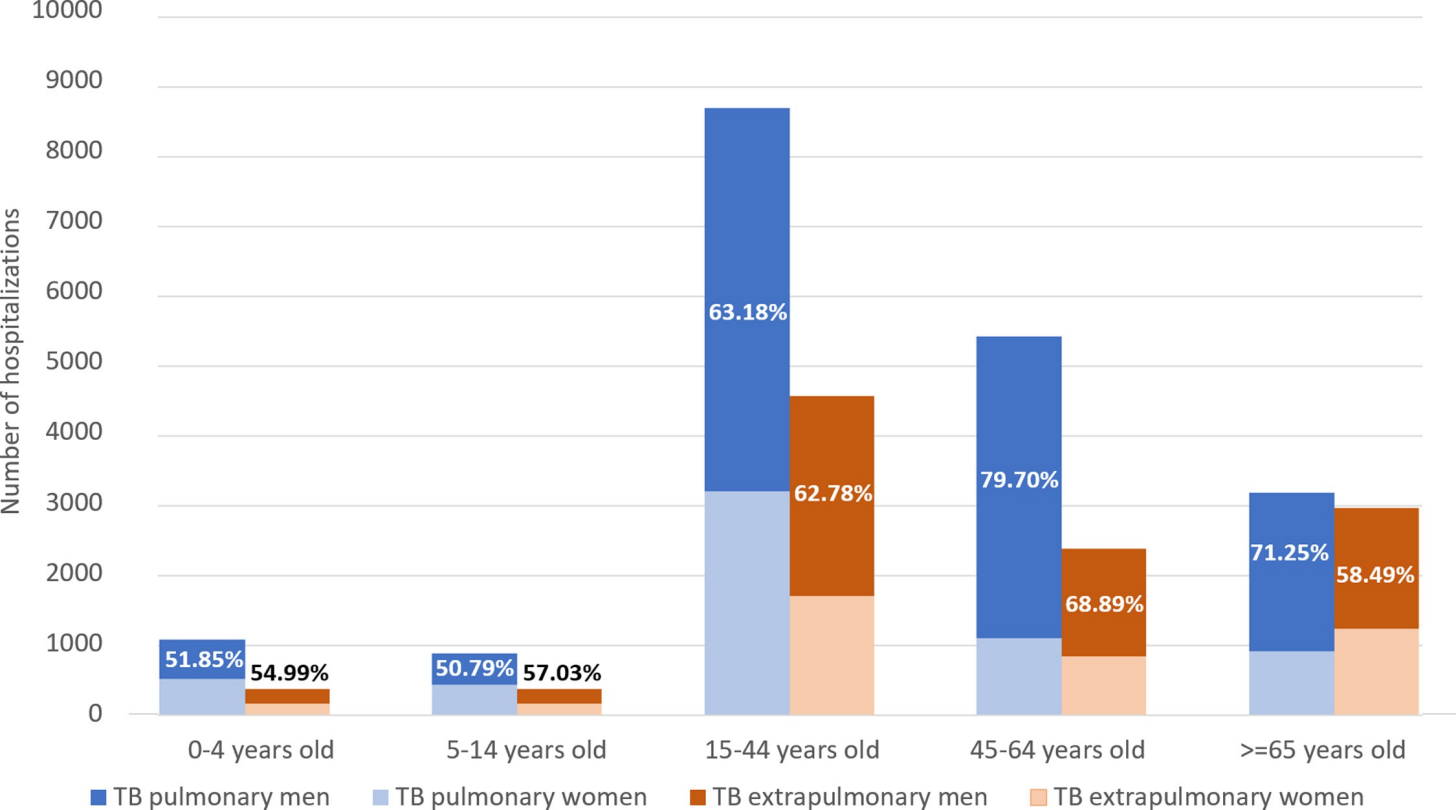
Number of hospitalizations by sex, age and type of tuberculosis. Percentage distribution according to sex. Spain, 2012–2020.

A total of 29,942 TB hospitalizations were recorded in the CMBD during the study period. The annual average hospitalization rate was 7.1 admissions/100,000. The male to female ratio was 1.5 (rates of 9.5 and 4.8/100,000 population for men and women, respectively). Hospitalizations with pulmonary forms were almost twice than extrapulmonary forms (4.6 vs 2.5/100,000). Regarding sex and type of TB, the frequency of pulmonary forms was higher among males (68% in males vs. 61.6% in females, p <0.001). The distribution of pulmonary forms varied by sex and age group. During childhood (<15 years), there were approximately the same number of males as females, while in the 45–64 age group males accounted for almost 80% of pulmonary cases (Fig 1).

The main age group was 15 to 44 years old, with a mean age of 44.3 years (standard deviation (SD) = 22.5). The mean age was lower in hospitalizations with pulmonary forms (mean 42.5 years; SD = 21.9) than in extrapulmonary forms (mean 47.5; SD = 23.3), this difference being statistically significant (p<0.001).

Mean age for pulmonary TB was 7 years lower in women than in men (37.8 years vs. 44.7 years, respectively), while for extrapulmonary forms, the mean age in women was one year above (48.1 and 47.2 years respectively, p<0.01). Males and those under 15 years of age presented more frequently pulmonary than extrapulmonary forms, with an odds ratio (OR) and adjusted odds ratio (ORa) of 1.4 and 1.6, respectively (95% CI 1.3–1.5; 95% CI 1.5–1.8) (Table 1).

**Table 1:** Characteristics of hospitalizations recorded in the CMBD and cases reported to RENAVE by type of tuberculosis. Spain, 2012–2020. A diagnosis of HIV was found in 872 records, which represents 2.9% of the total, being more frequent in the extrapulmonary forms than in the pulmonary ones. HIV-positive patients had a higher proportion of successive episodes (OR 1.6; 95% CI 1.4–1.9), but no more deaths were observed among these patients: of the 928 deaths, 22 involved HIV coinfection. Note: The disaggregated data count may differ from the total due to rounding and patients not included in any of the categories. The model was adjusted for pulmonary or not, age, sex, length of stay over 15 days, cost greater than the mean, successive episode, HIV and exitus.

Characteristics		PULM.	EXTRAPULM	OR	CI 95%	OR a	CI 95%
		n (%)	n (%)				
**Total**	CMBD	19,275 (64.37)	10,667 (35.63)				
	RENAVE	31,978 (71.83)	12,541 (28.17)				
**Sex male**	CMBD	13,097 (67.96)	6,568 (61.58)	1.32	1.26–1.39	1.39	1.32–1.46
	RENAVE	20,734 (64.84)	6,794 (54.17)	1.56	1.50–1.63	1.58	1.52–1.65
**Age <15 years**	CMBD	1,964 (10.19)	741 (6.95)	Ref		Ref	
	RENAVE	2,160 (6.75)	677 (5.40)	Ref		Ref	
**Age 15–64 years**	CMBD	14,129 (73.30)	6,956 (65.21)	0.77	0.70–0.84	0.74	0.67–0.81
	RENAVE	23,724 (74.19)	8,411 (67.07)	0.88	0.81–0.97	0.83	0.76–0.91
**Age >65 years**	CMBD	3,182 (16.50)	2,970 (27.84)	0.80	0.37–0.45	0.40	0.36–0.44
	RENAVE	6,092 (19.05)	3,453 (27.53)	0.55	0.50–0.61	0.52	0.47–0.57
**HIV**	CMBD	461 (2.39)	411 (3.85)	0.61	0.53–0.70	0.63	0.55–0.72
	RENAVE*	1,635 (8.21)	628 (8.17)	1.00	0.91–1.11	1.00	1.00–1.01
**Exitus**	CMBD	563 (2.92)	365 (3.42)	0.85	0.74–0.97	1.21	1.05–1.39
	RENAVE*	2,049 (9.96)	730 (8.53)	1.19	1.08–1.30	1.01	1.01–1.02
**Only in CMBD**							
**>15 days of stay**		5,790 (30.04)	3,873 (36.31)	0.75	0.72–0.79	0.84	0.80–0.89
**Cost >5710 €**		6,252 (32.44)	4,451 (41.73)	0.67	0.64–0.70	0.69	0.66–0.73
**Successive episode**		3,084 (16.00)	2,430 (22.78)	0.65	0.61–0.68	0.63	0.59–0.66
**Only in RENAVE**							
**Admission** *		9,962 (64.98)	3,851 (58.75)	1.30	1.23–1.38	1.02	1.01–1.02

*Note: percentages calculated from the total number of completed records, since HIV was included in 62% of the records, death in 65.4% and admission in 49.2%.

The mean hospital stay for all records was 16.2 days (SD = 30.5), and the median was 10 days (interquartile range (IR) = 5–19). Admissions for extrapulmonary forms lasted on average 1.5 days longer than those for pulmonary forms (17.2; SD = 22.1; p<0.01). In terms of costs, the mean was 5710.5 euros (€) (SD = 5044.2), and hospitalizations with extrapulmonary forms were significantly more expensive than pulmonary ones: mean 6592.5€ (SD = 6512.1) vs. 5222.4€ (SD = 3922.9), p<0.01.

A total of 18.4% of the episodes were considered “successive”, of which 27.2% were admitted two or more times. Pulmonary forms were associated with a lower frequency of successive episodes than extrapulmonary forms (ORa 0.6; 95% CI 0.6–0.7) (Table 1).

### Tuberculosis cases reported to RENAVE in Spain from 2012 to 2020

From 2012 to 2020, 44,520 cases of tuberculosis were reported to RENAVE, with a predominance of pulmonary forms (71.8%) and men (61.8%). The mean age was 45.7 years (SD = 21.5), three years higher in men than in women [(46.93 (SD = 20.94) vs. 43.8 years (SD = 22.4), p<0.05].

Information on exitus (yes/no) was filled in almost two thirds of the records (n = 29,130), out of which 9.5% (2,779 persons) died. HIV information was recorded in 62% registers and when compared by type of tuberculosis, the differences were not significant.

Pulmonary forms were more frequent in children under 15 years of age than extrapulmonary forms (p<0.01). This association was stronger when adjusted for other variables (ORa = 1.9; 95% CI: 1.5–2.3). Being male was also strongly associated with having a pulmonary form (ORa 1.6; 95% CI 1.5–1.6). Admission was recorded in 49.2% of the registries (n = 21,887), out of which 63.1% (n = 13,813 patients) were hospitalized. Patients with pulmonary forms were more likely to be admitted (OR 1.3; 95% CI 1.2–1.4), and to die (OR 1.2; 95% CI 1.1–1.3) than those with extrapulmonary forms, but when considering age and sex, as well as HIV, this statistical association was no longer significant (Table 1).

### Differences between CMBD and RENAVE

Table 2 summarized the main differences between both databases. Only not successive episodes from the CMBD (n = 24,428) were included. In both registries men prevailed, but the proportion in CMBD was slightly higher than in RENAVE (64.8% vs. 61.8%, p<0.001). RENAVE collected a higher percentage of pulmonary forms than CMBD (71.83% vs. 66.28%, p<0.001). By age group, the total cases in children under 5 years of age were very similar in both information systems, reflecting the high frequency of hospitalization in this group. As for HIV, the proportion of co-infections was almost four times higher in RENAVE than in CMBD.

**Table 2:** Patients with tuberculosis in the CMBD (excluding successive episodes) and cases registered in the RENAVE. Spain, 2012–2020.

Characteristics (n, (%))		CMBD non-successive (n = 24,428)	RENAVE (n = 44,520)	p
**Sex**	Male	15,829 (64.8)	27,528 (61.83)	<0.001
	Female	8,597 (35.2)	16,987 (38.16)	
**Age group (years),**	0–4	1,091 (4.47)	1,391 (3.12)	<0.001
	5–14	972 (3.98)	1,446 (3.25)	
	15–44	11,116 (45.51)	20,045 (45.05)	
	45–64	6,248 (25.58)	12,086 (27.15)	
	>65	5,001 (20.47)	9,546 (21.44)	
**Pulmonary**	No	8,237 (33.72)	12,541 (28.17)	<0.001
	Yes	16,191 (66.28)	31,979 (71.83)	
**HIV** *	No	23,789 (97.38)	25,332 (91.79) *	<0.001
	Yes	639 (2.62)	2,264 (8.20)	
**Exitus** *	No	23,592 (96.58)	26,358 (90.46) *	<0.001
	Yes	836 (3.42)	2,779 (9.54)	

*Note: percentages calculated from the total number of completed records, since HIV was included in 62% of the records, death in 65.4% and admission in 49.2%.

According to CMBD and RENAVE, there were 836 and 2,591 TB deaths from 2012 to 2020, respectively, although RENAVE data for 2020 were not consolidated at the time of the present study. The proportion of deaths was significantly higher in RENAVE than that recorded in CMBD (6.2% vs. 3.4%, respectively, p<0.001) (Table 2). During the same period, the INE recorded 2,290 deaths due to tuberculosis [16].

### Temporal evolution of tuberculosis according to CMBD and RENAVE

During the study period, a downward trend was observed for both hospitalizations and notifications, and also for both pulmonary and extrapulmonary forms.

In CMBD the decline was sharper until 2015, when the slope softened until 2018. In 2019 there was a small upturn in hospitalizations, decreasing again in 2020. The coefficient of determination (R2) was 0.8 (p<0.01) and the APC was -4.3 (p<0.05), with no significant change in trend identified.

**Fig 2:**
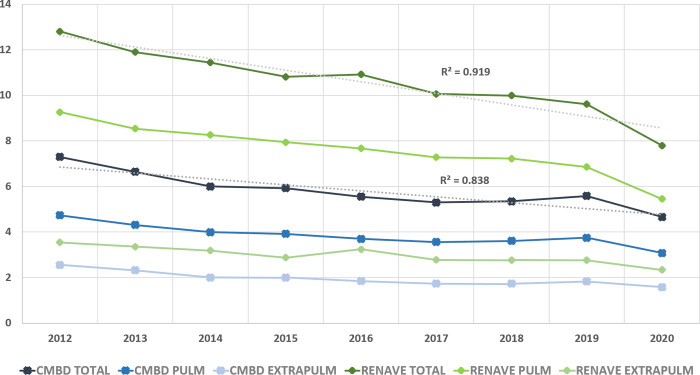
Evolution of tuberculosis rates per 100,000 population in RENAVE and CMBD by type of tuberculosis. Spain, 2012–2020.

Note: Although RENAVE data for 2019 and 2020 were not consolidated at the time of the study, they were maintained for the purpose of temporal comparison with CMBD.

In RENAVE, the trend was downward from 2012 to 2020, except for a small rebound in 2016 (R2 = 0.9; p<0.01). In the Jointpoint regression analysis, the APC was -4.82 (p<0.05), with no significant trend change. The analysis was repeated without 2019–20 data, as the data for some CCAA were incomplete, showing a correlation coefficient of 1 (p<0.01) and an APC of -3.7 (p<0.05) (Fig 2).

**Fig 3:**
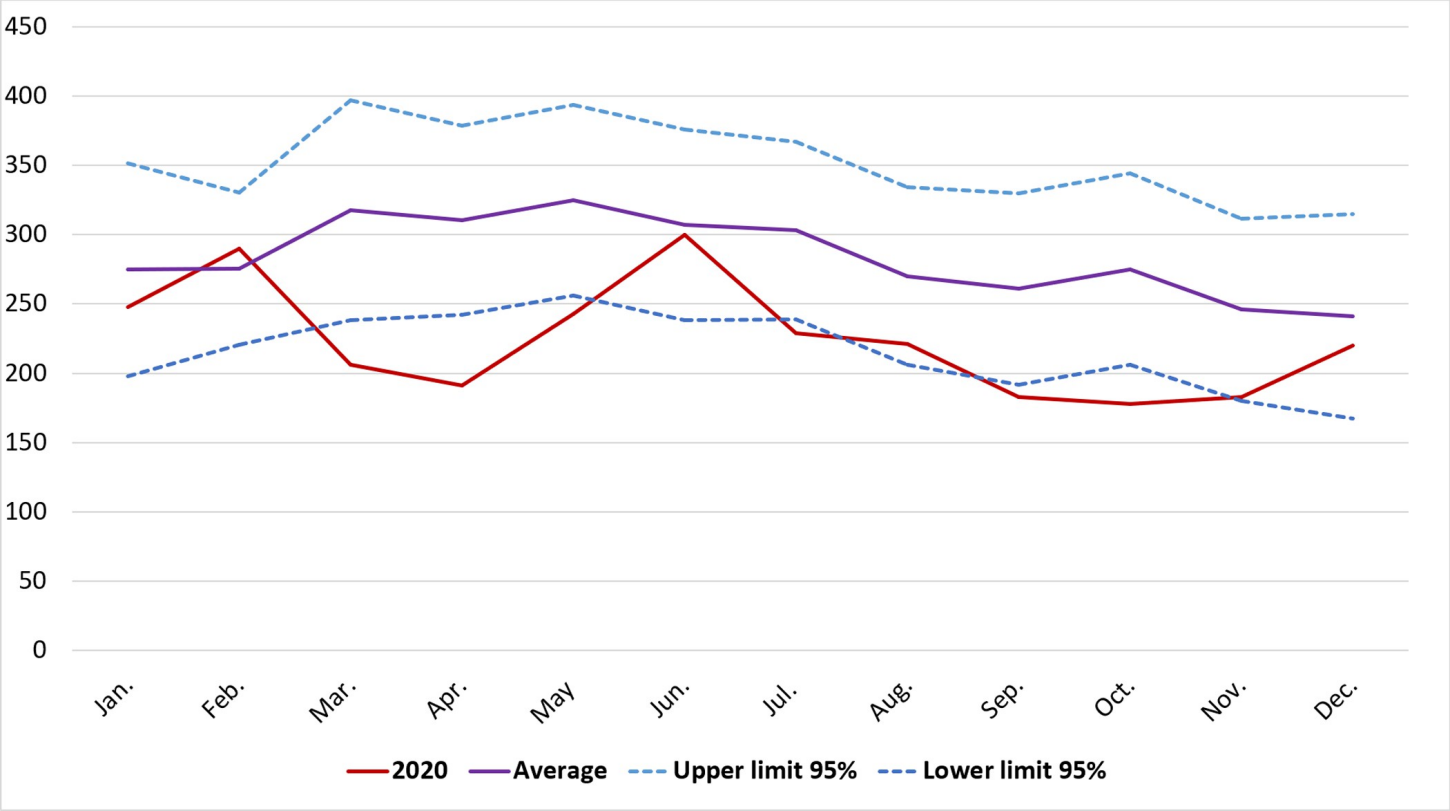
Temporal analysis of monthly moving averages of hospitalized cases due to tuberculosis from 2012 to 2019 compared to 2020 in Spain.

Analysis of monthly hospitalizations throughout 2020, compared to the monthly moving averages of the previous 8 years, showed a significant decline in TB hospitalizations (Fig 3). January and February were similar to the previous period, but during the following three months, the number of hospital admissions were below the lower limit. In June, with 300 hospitalizations, the monthly average was almost reached (307; SD = 35.0), but from September to October, admissions again fell below the expected level. The year ended with a slight increase in hospitalizations.

### Spatial trends in tuberculosis according to the CMBD and the RENAVE records

**Fig 4:**
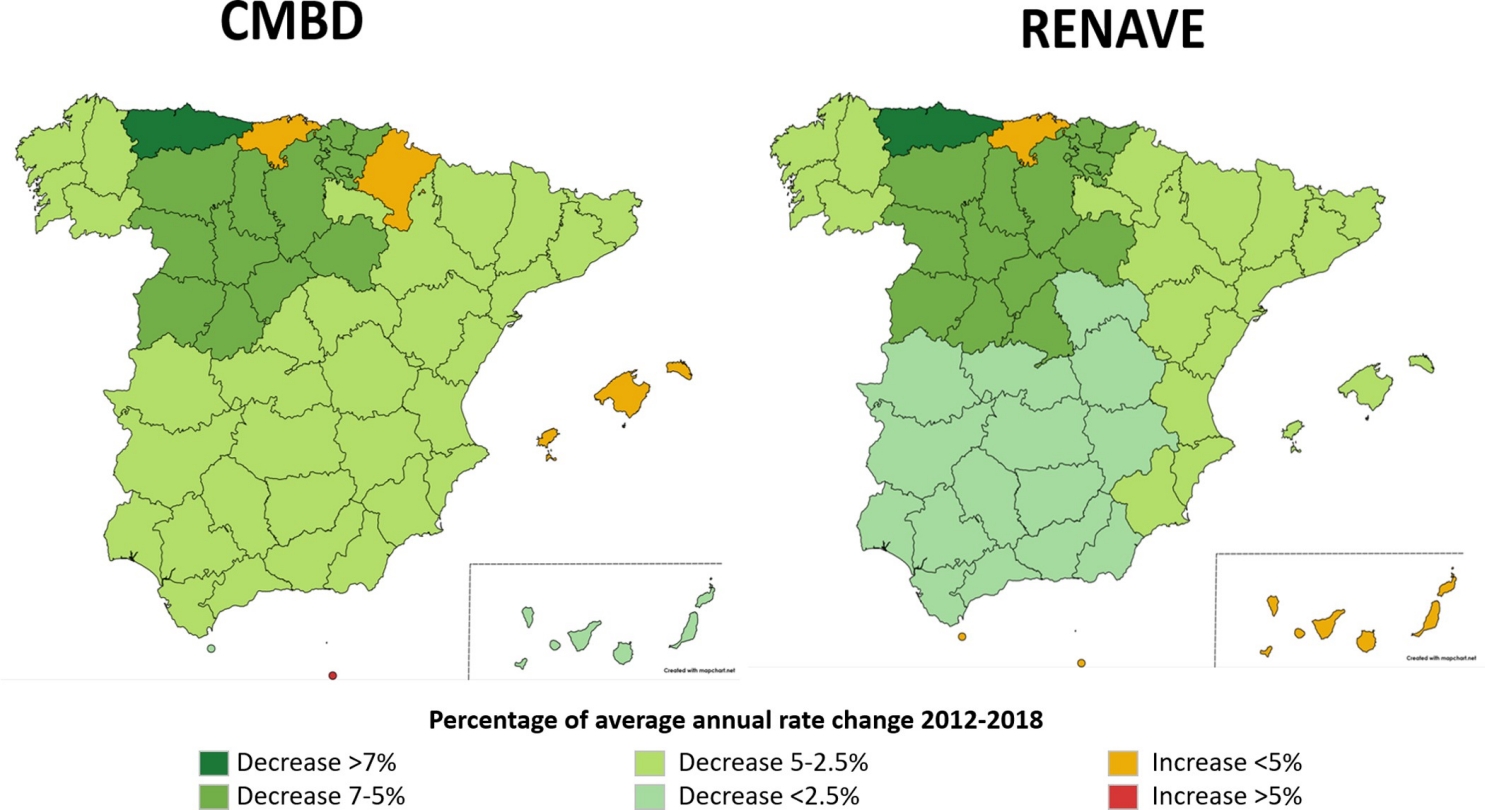
Annual tuberculosis rate changes. Spain, 2012–2018.

Annual rates per 100,000 population and CCAA are shown in S2 and S3 Tables. Almost all the CCAA shown a downward trend over the study period (Fig 4). Asturias, in particular, showed the greatest decrease in both notifications and hospitalizations (more than 7%). Declines between 5% and 7% were observed in Castilla and Leon, the Basque Country and Madrid, the latter only according to data from RENAVE. Only Melilla recorded a significant increase in the number of TB hospitalizations per 100,000 inhabitants during the study period (26.7%), while figures remained practically stable according to RENAVE. There was no increase of more than 5% in RENAVE for any of the CCAA.

Between both systems, the differences in annual rate change trends were generally less than 3%. Only four CCAA exceeded this percentage difference; Melilla, Balearic Islands and Navarre showed an increase in the annual rate recorded in the CMBD, whereas in RENAVE this rate decreased. In contrast, in Castilla-La Mancha there was a greater decrease in hospitalizations than in notifications (Table 3).

**Table 3:** Average annual rate change. Spain, 2012–2018.

	RENAVE	CMBD
ANDALUSIA	-1.01%	-3.29%
ARAGON	-4.00%	-4.70%
ASTURIAS	-8.45%	-8.74%
BALEARIC ISLANDS	-2.95%	4.66%
CANARY ISLANDS	2.50%	-0.17%
CANTABRIA	3.66%	0.74%
CASTILLA-LA MANCHA	-0.56%	-4.98%
CASTILLA AND LEON	-6.82%	-6.64%
CATALONIA	-3.34%	-4.41%
CEUTA	0.29%	-0.52%
VALENCIA	-4.15%	-4.11%
EXTREMADURA	-2.12%	-4.21%
GALICIA	-3.26%	-3.98%
LA RIOJA	-4.62%	-3.87%
MADRID	-5.74%	-3.17%
MELILLA	0.76%	26.63%
MURCIA	-2.60%	-3.11%
NAVARRE	-3.63%	1.31%
BASQUE COUNTRY	-6.87%	-6.13%
Total	-3.99%	-4.97%

## Discussion

Through the study of CMBD and RENAVE, a broad and complementary picture of the epidemiological situation of tuberculosis in Spain, from 2012 to the COVID-19 pandemic in 2020, is presented. To our knowledge, this is the first time that both databases are analyzed together to refine the epidemiological description of a disease that, despite its progressive decrease, continues to be a relevant public health problem in this country.

According to our results, there is a higher proportion of TB cases among males. This difference has been observed for years and in all countries, although it seems to be attenuated in the most extreme ages of life [19–22]. Several hypotheses are proposed, such as a major chance of underdiagnosis in women [20], a higher risk of exposure or certain biological characteristics that increase their vulnerability, like differences in the formation of pulmonary B cell follicles [22].

Approximately 45% of the TB patients attending specialized health units or reported to RENAVE were in the age group of 15 to 44 years, a slightly higher percentage than expected according to the national epidemiological reports [11], but probably related to the fact that both domestic and imported were analyzed. Globally, the WHO also estimates that the highest incidence rates are found in this age group, starting a decline from 35 years for women and 55 for men [1, 23]. In fact, adolescents are a key target group for TB control interventions, since, as stated by the WHO, adolescents are more often contagious and have a larger number of contacts in closed spaces such as schools [24]. It is also remarkable that children under 5 years of age were the only age group in which the percentage of hospitalizations exceeds that of reported cases, probably because children, especially those under 2 years of age, have more acute symptoms with more severe forms and a higher complication rate [25].

In our study, those over 65 years old were the third group in frequency. In this age group extrapulmonary forms were more frequent, as found in a retrospective cohort of more than 1,000 patients in the United Kingdom [26]. Moreover, hospitalized patients with this kind of locations presented a greater complexity (more age, coinfection with HIV, several successive episodes, longer stays, and more deaths), probably in relation to the need to rule out concomitant pulmonary tuberculosis [27, 28]. As Seersholm and Wilcke showed in their study, diagnosing extrapulmonary tuberculosis is still very challenging [29]. On the contrary, cases reported to RENAVE with pulmonary TB shown greater risk of being hospitalized and die (OR = 1.3 and 1.2 respectively, Table 1). This difference between both data sources may be due, among other reasons, to the fact that extrapulmonary forms may be underreported in RENAVE due to the greater diagnostic difficulty or because of a higher follow-up complexity [30, 31]. For this reason, it is essential to increase the diagnostic suspicion for these kind of TB forms, especially among the elderly [9].

TB/HIV coinfection rates were lower in CMBD than in RENAVE, but this is probably related to the fact that it is not mandatory to fulfill the HIV status in CMBD records. According to the European "Tuberculosis Annual Epidemiological Report", there is a 3.1% of coinfection rate among those countries with comprehensive data, quite lower that the rate provided by RENAVE (8.2%).

Regarding fatal outcome, the chances of a patient dying increased among those over 44 years old, and HIV co-infection, according to both data sources [32]. No differences were observed by clinical form. In RENAVE, hospitalization was associated with an increased risk of dying, which can be explained by two facts: if a patient dies, he is more likely to have been previously hospitalized, and patients with HIV and older are more likely to die during hospitalization [32, 33].

### Temporal trends

The average rate of TB related hospitalizations during the study period was almost half the one observed between 1999 and 2009 (13.9/100000) [10]. This downward trend, also shown by the RENAVE data, is in line with the rest of EU countries, approaching the goal of UN SDGs, but not fast enough to achieve the elimination in the region by 2030 [6]. Moreover, success treatment rates in this region, both in sensitive and resistant TB forms, are still much lower than the committed objectives [19, 34].

The public health efforts made in TB control during the last two decades are reflected in this downward trend, especially with regard to pulmonary TB [35], which generally corresponds to incident cases. On the other hand, extrapulmonary forms tend to correspond to prevalent cases, in older, longer-infected patients with more insidious presentations [28]. Therefore, the impact of control measures may need longer to be observed and assessed.

It is also important to consider the impact of changes in the reporting criteria throughout the study period; i.e., CMBD was extended to all forms of specialized care in 2016, including outpatient care [14], without correlating with a significant change in trend, but with a smaller decrease in new cases until 2019, when a slight increase was objectified. In RENAVE, a slight rebound of cases was observed in 2016, mainly due to extrapulmonary forms, without being associated with a significant change in trend. This upturn is observed in other European countries [34], but is not detected in CMBD.

The COVID-19 pandemic and its consequences, both in terms of health and border closures, marked 2020 as a particular year. When analyzing the monthly hospitalization rates and comparing them with the previous 8 years, seasonal changes are noticeable up to 2019: in spring and summer there used to be an increase in the number of cases [36]. However, in 2020 this situation was reversed, with decreases in spring and autumn, coinciding with the initial lockdown, and the first two waves of the COVID-19 pandemic in Spain [37], which peaked in late March and October, respectively. The COVID-19 pandemic has caused enormous health, social and economic impacts in 2020 and 2021 all around the world. This includes impacts on the provision of and access to TB services, the number of people diagnosed/notified with TB through national disease surveillance systems, among others [1, 38]. These disruptions may have impacted not only the disease seasonality but TB disease burden (in terms of incidence and mortality). Morevover, data from the last Global Tuberculosis Report 2020 suggest that other impacts associated with the COVID-19 pandemic include a decline in people enrolled on treatment for MDR/RR-TB; a downturn in the number of people initiated on TB preventive treatment; and a reduction in spending on TB prevention, diagnostic and treatment services. Although, figures seem to be similar in different countries, further analysis are needed for contextualization, as some structural factors may differ. Moreover, expanded data on post-pandemic period will be key for a better assessment.

### Geographical distribution

In general terms, the trend was downward or with very insignificant increases in both registries. Only Melilla showed a relevant increase in the number of hospitalizations. Migratory flows, its geographical location and the demographics of this autonomous city could explain this fact. The last epidemiological report on tuberculosis in Spain [2] found that 28.7% of the reported cases were born in another country, with Morocco being the most frequent country of birth. It should be noted that, when analyzing age by CCAA, only in Ceuta and Melilla the average age was below 5 years, in accordance with their population pyramid, context and geographic location [16, 39].

Migratory movements could help to explain the decline observed in Asturias and Castilla and Leon. According to the INE [16], they were among the CCAA that have experienced the greatest depopulation in recent years. Furthermore, these communities recorded the highest mean ages of the cases, above 56 years of age.

## Limitations and conclusions

When working with such different and broad sources of information as RENAVE and the CMBD, it is essential to make some decisions that may affect the results. In the CMBD, by considering only the main diagnosis, information was lost on the cases with both types of TB, which could explain why the percentage of pulmonary forms detected was lower than that collected in RENAVE (71.8% vs. 66, 3% in the CMBD). Information on deaths was also lost, when the deceased did not have TB in the first diagnostic position at admission. To confirm this fact, the deaths in the total CMBD records with TB in any position were analyzed, observing ten times more than if only the first diagnosis was considered (8,565).

Regarding RENAVE, approximately 20,000 more cases were observed than in CMBD, something to be expected, since not all patients diagnosed with TB are hospitalized, but all those admitted with active disease should be notified [12, 14]. The completion of some variables was low, especially “admission” (contained in 13,913 records), “discharge”, “mortality” and “HIV”. In the last available RENAVE report from 2020 [2], the serological HIV status was missing in 30% of the cases, something that is also observed in the ECDC and WHO reports [1, 34].

This information bias could be influenced by the type of tuberculosis: pulmonary forms, which have been notifiable for a longer time [40], and require more follow-up due to the study of contacts, were more exhaustively reported.

Finally, it is noteworthy the limitation for the temporal analysis that some CCAA had not consolidated their RENAVE data for 2019 and 2020, which shortened the observed period, distancing it from the current situation.

Despite its limitations, the joint analysis of both information systems favors knowledge of TB in Spain. The CMBD is not designed as a surveillance system, but its information may improve the completeness of the registries, as it has already been proven with other diseases [6, 34].

Otherwise, hospitalizations could provide more immediate information on disease status, without the delays resulting from the need to consolidate information. The 2020 monthly analysis in the CMBD supports the hypothesis that the COVID-19 pandemic has affected tuberculosis in multiple ways [1, 17] and in the coming years we might observe the consequences, with increased notifications and possible increased mortality. 2019–20 information from the surveillance system is more difficult to explain and even presents results that might be contrary to this hypothesis. This is due to the delay in the consolidation of data, which is especially relevant in the case of TB as the information need to be updated at least every 6 months. Moreover, this delay (and the overall quality of data) got worst during pre-pandemic year 2019 and pandemic year 2020 due to the work overload at the national and regional public health services. Coming RENAVE data for 2021 and 2022 (when available) will help us to better interpret this temporal pattern and to give informed recommendations.

Increased knowledge and enhanced use of all information systems are necessary to complement surveillance systems, to compensate for registries’ limitations, and in order to fill information gaps, and to implement strategies using resources in the most efficient way. All means are necessary to join efforts towards the control of this long-known, curable and preventable disease.

## Supporting information

S1 Table ICD-9 and ICD-10 codes for pulmonary and extrapulmonary TB. (DOCX)

S2 Table Annual rate per 100,000 population of non-successive TB hospitalizations in CMBD by CCAA. Spain, 2012–2020.
(DOCX)

S3 Table Annual rate per 100,000 population of cases reported to RENAVE by CCAA. Spain, 2012–2020.
(DOCX)
