# Supplementary material for: The epidemiological situation of tuberculosis in Spain according to surveillance and hospitalization data, 2012–2020
Source: PLoS One. 2024 Jan 2;19(1):e0295918. doi: 10.1371/journal.pone.0295918 (PMC10760747; doi:10.1371/journal.pone.0295918)
Supplement: S1 Table — (DOCX) [file pone.0295918.s001.docx]

|  | **PULMONARY**  **TUBERCULOSIS** | | | | **EXTRAPULMONARY**  **TUBERCULOSIS** | | | | | | |
| --- | --- | --- | --- | --- | --- | --- | --- | --- | --- | --- | --- |
| **ICD-9** | 010.0  010.8  010.9  011.0  011.1  011.2 | 011.3  011.4  011.5  011.6  011.7  011.8 | 011.9  012.2  012.3  012.8 | 013.0  013.1  013.2  013.3  013.4  013.5  013.6 | | 013.7  013.8  013.9  014.0  014.8  015.0  015.1 | 015.2  015.5  015.6  015.7  015.8  015.9  016.0 | 016.1  016.2  016.3  016.4  016.5  016.6  016.7 | 016.8  016.9  017.0  017.2  017.3  017.4  017.6 | 010.1  017.7  017.8  017.9  018.0  018.8  018.9 | 012.0  012.1  795.51  V12.01  137 |
| **ICD-10** | A15.1  A15.2  A15.3  A15.0  A15.5 | A15.7  A15.8  A15.9  A16.0  A16.1 | A16.2  A16.4  A16.8  A16.9  A17.7 | A15.4  A15.6  A16.3  A16.5 | | A17.0  A17.1  A17.8  A17.9 | A18.0  A18.1  A18.2  A18.3  A18.4 | A18.5  A18.6  A18.7  A18.8  A19.0 | A19.1  A19.2  A19.8  A19.9  J65 | Z86.11  Z86.15  Z22.7  R76.11  R76.12 | B90.0  B90.1  B90.2  B90.8  B90.9 |
