# Supplementary material for: The epidemiological situation of tuberculosis in Spain according to surveillance and hospitalization data, 2012–2020
Source: PLoS One. 2024 Jan 2;19(1):e0295918. doi: 10.1371/journal.pone.0295918 (PMC10760747; doi:10.1371/journal.pone.0295918)
Supplement: S2 Table — Spain, 2012–2020. (DOCX) [file pone.0295918.s002.docx]

Supplementary Table 2 Annual rate of non-successive TB episodes in CMBD by CCAA. Spain, 2012-2020

|  | **2012** | **2013** | **2014** | **2015** | **2016** | **2017** | **2018** | **2019** | **2020** | **Mean** |
| --- | --- | --- | --- | --- | --- | --- | --- | --- | --- | --- |
| ANDALUSIA | 5.59 | 5.27 | 5.20 | 5.44 | 4.52 | 4.31 | 4.50 | 4.23 | 3.42 | 4.72 |
| ARAGON | 8.95 | 10.64 | 8.73 | 8.32 | 6.30 | 7.45 | 6.08 | 6.19 | 5.63 | 7.60 |
| ASTURIAS | 10.55 | 10.63 | 9.30 | 7.09 | 8.10 | 5.53 | 5.57 | 5.00 | 6.99 | 7.67 |
| BALEARIC ISLANDS | 8.24 | 6.47 | 8.03 | 6.37 | 2.54 | 5.01 | 5.44 | 6.01 | 5.60 | 5.95 |
| BASQUE COUNTRY | 8.62 | 7.32 | 7.16 | 6.15 | 6.65 | 5.77 | 5.75 | 5.87 | 5.71 | 6.56 |
| C. AND LEON | 8.47 | 7.10 | 5.79 | 5.92 | 5.93 | 4.04 | 5.02 | 5.70 | 5.14 | 5.92 |
| C.-LA MANCHA | 5.48 | 4.85 | 3.87 | 3.02 | 3.18 | 3.49 | 3.79 | 3.09 | 2.50 | 3.70 |
| CANARY ISLANDS | 3.44 | 2.70 | 2.97 | 3.24 | 2.71 | 2.22 | 3.02 | 3.15 | 3.16 | 2.96 |
| CANTABRIA | 7.27 | 6.63 | 7.51 | 6.68 | 7.57 | 7.40 | 7.40 | 6.02 | 4.29 | 6.75 |
| CATALONIA | 7.78 | 7.43 | 6.84 | 5.94 | 5.53 | 6.29 | 5.80 | 6.60 | 5.51 | 6.41 |
| CEUTA | 23.75 | 18.93 | 20.09 | 18.94 | 17.70 | 7.06 | 12.95 | 20.13 | 14.29 | 17.09 |
| EXTREMADURA | 7.89 | 4.92 | 4.75 | 4.69 | 4.35 | 4.47 | 5.44 | 4.89 | 2.74 | 4.92 |
| GALICIA | 10.20 | 9.48 | 7.08 | 8.51 | 6.82 | 6.51 | 7.34 | 7.41 | 5.89 | 7.70 |
| LA RIOJA | 9.06 | 8.84 | 5.41 | 5.11 | 7.04 | 6.40 | 6.08 | 3.50 | 2.85 | 6.04 |
| MADRID | 7.58 | 6.12 | 5.68 | 6.51 | 6.90 | 5.86 | 5.97 | 6.60 | 4.56 | 6.19 |
| MELILLA | 12.10 | 17.93 | 21.41 | 18.90 | 7.08 | 21.22 | 14.18 | 22.51 | 17.79 | 17.02 |
| MURCIA | 8.35 | 7.66 | 7.59 | 6.97 | 4.83 | 5.56 | 6.36 | 7.36 | 6.43 | 6.79 |
| NAVARRE | 6.57 | 5.18 | 6.45 | 5.50 | 3.60 | 4.06 | 5.73 | 1.84 | 2.43 | 4.58 |
| VALENCIA | 6.50 | 6.62 | 4.93 | 5.35 | 6.53 | 5.68 | 4.63 | 5.46 | 4.82 | 5.61 |
| Total | **7.30** | **6.64** | **6.00** | **5.93** | **5.55** | **5.30** | **5.35** | **5.59** | **4.66** | **5.81** |
