# Supplementary material for: The epidemiological situation of tuberculosis in Spain according to surveillance and hospitalization data, 2012–2020
Source: PLoS One. 2024 Jan 2;19(1):e0295918. doi: 10.1371/journal.pone.0295918 (PMC10760747; doi:10.1371/journal.pone.0295918)
Supplement: S3 Table — Spain, 2012–2020. (DOCX) [file pone.0295918.s003.docx]

Supplementary table 3 Annual rate per 100,000 population of cases reported to RENAVE by CCAA. Spain, 2012-2020.

|  | **2012** | **2013** | **2014** | **2015** | **2016** | **2017** | **2018** | **2019** | **2020** | **Mean** |
| --- | --- | --- | --- | --- | --- | --- | --- | --- | --- | --- |
| ANDALUSIA | 9.17 | 8.57 | 8.62 | 8.10 | 8.46 | 7.97 | 8.55 | 8.08 | 6.52 | 8.23 |
| ARAGON | 11.93 | 13.71 | 12.80 | 10.89 | 12.99 | 10.34 | 8.66 | 10.12 | 6.31 | 10.86 |
| ASTURIAS | 18.31 | 15.81 | 16.03 | 12.16 | 13.02 | 10.97 | 10.35 | 11.08 | 12.21 | 13.33 |
| BALEARIC ISLANDS | 12.95 | 10.87 | 11.87 | 9.12 | 9.62 | 11.23 | 10.12 | 6.34 | 6.09 | 9.80 |
| BASQUE COUNTRY | 15.83 | 14.79 | 14.92 | 11.84 | 12.70 | 10.52 | 9.99 | 9.07 | 10.60 | 12.25 |
| C. AND LEON | 13.77 | 11.45 | 11.27 | 9.90 | 10.31 | 8.95 | 8.84 | 8.62 | 7.15 | 10.03 |
| C.-LA MANCHA | 8.15 | 7.92 | 8.13 | 6.52 | 8.22 | 6.84 | 7.34 | 0.05 | 0.00 | 5.91 |
| CANARY ISLANDS | 7.17 | 7.68 | 6.14 | 7.57 | 5.93 | 5.96 | 7.49 | 6.08 | 5.17 | 6.58 |
| CANTABRIA | 11.50 | 12.40 | 11.26 | 10.11 | 11.52 | 10.33 | 13.43 | 8.08 | 7.56 | 10.69 |
| CATALONIA | 16.29 | 15.68 | 15.31 | 14.64 | 13.52 | 13.38 | 13.26 | 14.38 | 11.45 | 14.21 |
| CEUTA | 42.76 | 20.12 | 35.45 | 21.31 | 35.41 | 10.59 | 12.95 | 20.13 | 1.19 | 22.21 |
| EXTREMADURA | 8.26 | 7.28 | 5.94 | 6.07 | 6.57 | 5.40 | 6.75 | 4.99 | 1.98 | 5.92 |
| GALICIA | 24.08 | 21.76 | 20.37 | 21.65 | 20.83 | 19.42 | 19.56 | 19.19 | 14.67 | 20.17 |
| LA RIOJA | 13.43 | 15.15 | 10.19 | 8.63 | 10.88 | 9.60 | 8.95 | 9.22 | 6.96 | 10.34 |
| MADRID | 12.82 | 10.98 | 10.05 | 10.47 | 10.77 | 9.32 | 8.84 | 8.63 | 7.04 | 9.88 |
| MELILLA | 19.36 | 26.30 | 45.20 | 30.71 | 22.43 | 21.22 | 13.00 | 14.22 | 10.68 | 22.57 |
| MURCIA | 11.02 | 10.06 | 9.02 | 9.97 | 7.83 | 8.28 | 8.99 | 9.30 | 8.15 | 9.18 |
| NAVARRE | 12.20 | 8.63 | 10.53 | 7.86 | 6.58 | 6.55 | 8.36 | 4.90 | 4.87 | 7.83 |
| VALENCIA | 10.04 | 10.23 | 9.26 | 8.84 | 9.84 | 9.23 | 7.58 | 9.34 | 6.69 | 9.01 |
| Total | **12.80** | **11.90** | **11.44** | **10.82** | **10.92** | **10.07** | **9.99** | **9.62** | **7.80** | **10.60** |
